# Polyhydramnios, or Dropsy of the Amnion

**Published:** 1902-01

**Authors:** V. P. Smith

**Affiliations:** Jamestown, Ohio


### POLYHYDRAMNIOS, OR DROPSY OF THE AMNION.

By V. P. Smith, V.S.,
JAMESTOWN, OHIO.

(This subject is one of which I have no literature, and did not hear mentioned at the Ontario Veterinary College, where I was a student in 1891–92, or at the Chicago Veterinary College, from which I graduated in 1895.)

I was called, in April, to treat a case which may be of interest to some other practitioner, and an outline of the same may be of some service. The subject was a mare due to foal in May, which the owner said was getting so heavy that she could hardly walk, and he thought there was something wrong. On this visit I found the mare presenting symptoms of abdominal ascites, with belly very much dropped, and œdematous swelling along the abdomen, thorax, and between the forelegs. Appetite impaired; pulse, 60; temperature, 100. She presented the appearance of a case of ascites, and had I not examined per vaginam I would have diagnosed it as such; but on entering the hand into the vagina I found the cervix dilated and the placental membrane starting to protrude. I wanted to deliver the mare of the colt at once, but the owner objected, saying it was a month too soon, and it might kill her, so I left some medicine to be given that I was sure would do no harm, and told him to report to me if she got much larger, or if she showed signs of labor pains. I heard nothing more from her for two weeks, when he came for me to go and see her again. On my arrival this time I found the mare down; breathing very labored, pulse 90 and weak, temperature subnormal. We helped her to her feet, but it was with difficulty that we kept her up until I could make an examination. She was as full of fluid as her hide would hold; was down until her abdomen was straight from knees to hocks. She did not look like a horse at all. She was all body. On inserting the hand in the vagina I found the cervix obliterated, the amniotic membrane distended and filling the pelvic cavity to its utmost capacity. When I first touched this membrane I thought it was the body of the uterus, but as I could feel no os I soon changed my mind. Finding the membrane too strong to rupture with my hand, I used a knife to make an opening; this allowed the fluid to run out to a level with the floor of the pelvis. When this was done I inserted my hand to see if I could get the colt, and I found it lying so far down that I could just barely touch it with my fingers, and it felt as though it were lying in the bottom of a large hogshead of water, and evidently dead. At this point I allowed the mare to lie down again and the remainder of the fluid to run out, and I feel safe in saying that there was at least forty gallons of it, and probably more. When this had mostly run out I proceeded to deliver the colt, which was easy after removal of the fluid. The mare looked like a collapsed balloon, her sides falling almost together. I used antiseptic and astringent wash in uterus, and gave stimulants, but the mare was unable to rise, and died in about forty-eight hours. I feel sure that if I had taken the colt on my first visit that I could have saved the mare, although she was nineteen or twenty years old; but I would advise all practitioners, when called to see a pregnant animal showing signs of dropsy or ascites, to examine per vaginam, and if they find the cervix dilated and the amniotic membrane protruding, proceed to deliver at once; if you cannot save the colt, you can save the mare. I do not consider it unsafe to deliver a mare of her colt any time after ten months, and I frequently do when I find them carrying the colt overtime and making no preparation for foaling, and always save both mare and colt.

If any reader desires anything further on this subject I would gladly answer all questions, either by letter or through the JOURNAL.

---

Dr. George R. White, Columbian University, '97, and Dr. Joseph Plaskett, McGill University, '92, have formed a partnership under the firm name of White & Plaskett. They have just completed the erection of the Nashville Veterinary Hospital, which is probably the largest as well as the best equipped veterinary hospital in the South. Dr. White continues to serve as City Veterinarian of Nashville.

Dr. J. Barton, of Washington, D. C., has gone to Pasadena, Cal., where he will continue to practice his profession.

